# New publication

**DOI:** 10.1155/S1463924601000189

**Published:** 2001


					The information will be of particular interest to engin-
eers, risk assessors and safety professionals in the chemical
industry who have to deal with water reactive chemicals.
Copies of REACTPOOL: a new Model for Accidental
Releases of Water Reactive Chemicals (CRR331/2001),
can be ordered from http://www.hsebooks.co.uk or from HSE
Books, PO Box 1999, Sudbury, Suolk CO10 2WA, UK.
Tel: + 44 (0)1787 881165; Fax: 01787 313995
New publication
Pharmaceutical Substances
A new revised and expanded 4th edition of Pharma-
zeutische Wirkstoe by A. Kleemann and J. Engel has
been published in English as Pharmaceutical Substances:
Bibliography. It contains information on over 2200 phar-
maceutical compounds of interest to the chemical and
pharmaceutical industry. It is available in 2001 on CD-
ROM for DM1498.00/O
 S10935.00/SF1318.00, ISBN 3-
13-115134-X/695.
134
Announcements/New publication

				

